# Efficient and Near-Zero Thermal Quenching Cr^3+^-Doped Garnet-Type Phosphor for High-Performance Near-Infrared Light-Emitting Diode Applications

**DOI:** 10.3390/molecules29174253

**Published:** 2024-09-07

**Authors:** Zaifa Yang

**Affiliations:** College of Physics and Electronic Engineering, Qilu Normal University, Jinan 250200, China; yangzaifa@qlnu.edu.cn; Tel.: +86-10-6677-8147

**Keywords:** NIR phosphors, Cr^3+^, near-zero thermal quenching

## Abstract

In recent years, near-infrared (NIR) phosphors have attracted great research interest due to their unique physical properties and broad application prospects. However, obtaining NIR phosphors with both high quantum efficiency and excellent thermal stability remains a great challenge. In this study, novel NIR Ca_3_Mg_2_ZrGe_3_O_12_:Cr^3+^ phosphors were successfully prepared using a high-temperature solid-phase method, and the structure and luminescent properties of the material were systematically investigated. Ca_3_Mg_2_ZrGe_3_O_12_:0.01Cr^3+^ emits NIR light in the range of 600 to 900 nm with a peak at 758 nm and a half-height width of 89 nm under the excitation of 457 nm blue light. NIR luminescence shows considerable quantum efficiency, and the internal quantum efficiency of the optimized sample is up to 68.7%. Remarkably, the Ca_3_Mg_2_ZrGe_3_O_12_:0.01Cr^3+^ phosphor exhibits a near-zero thermal quenching behavior, and the luminescence intensity of the sample at 250 °C maintains 92% of its intensity at room temperature. The mechanism of high thermal stability has been elucidated by calculating the Huang Kun factor and activation energy. Finally, NIR pc-LED devices prepared from Ca_3_Mg_2_ZrGe_3_O_12_:0.01Cr^3+^ phosphor with commercial blue LED chips have good performance, proving that this Ca_3_Mg_2_ZrGe_3_O_12_:0.01Cr^3+^ NIR phosphor has potential applications in night vision and biomedical imaging.

## 1. Introduction

Near-infrared (NIR) light sources are widely used in the fields of tissue imaging, security monitoring, iris recognition, and food detection [1,2]. At present, the traditional NIR light sources are halogen tungsten lamps, NIR light-emitting diodes, and infrared lasers [3]. However, the halogen tungsten lamp has the problem of a large volume and short life, the NIR light-emitting diode has the disadvantage of its half-height width and poor thermal stability, and the infrared laser is limited by its high cost and narrow spectral band [4]. In recent years, NIR phosphor-converted light-emitting diodes (NIR pc-LEDs) have attracted widespread attention from researchers due to their long lifespan, low cost, adjustable spectrum, small size, and energy-saving and environmentally friendly advantages [5]. The optical properties of NIR phosphors in NIR pc-LEDs determine the spectral distribution and performance advantages of these devices. Therefore, the development of high-performance broadband NIR phosphors has become a hot topic in the field of luminescent materials.

To achieve luminescence in the NIR-I and NIR-II regions, current ion doping strategies primarily focus on doping with rare earth (RE) ions and transition metal (TM) ions [6]. For instance, RE ion doping strategies utilize ions such as Er^3+^, Nd^3+^, Yb^3+^, Ho^3+^, Eu^2+^, and Tm^3+^, whereas TM ion doping strategies incorporate ions like Cr^3+^, Cr^4+^, Mn^2+^, Mn^4+^, and Ni^2+^ [7,8]. Additionally, co-doping strategies, including Er^3+^/Yb^3+^, Tm^3+^/Ho^3+^/Yb^3+^ and Cr^3+^/Ni^2+^, are being extensively studied to optimize luminescence performance. Trivalent chromium ions (Cr^3+^), notable among transition metal ions, have found extensive applications in advanced fields such as optical temperature sensors, photodynamic medical treatment, and full-spectrum lighting. Cr^3+^ ions are known to exhibit rich luminescent properties and a broad full width at half maximum (FWHM) under varying crystal field strengths provided by different host materials, owing to their unique 3D electron configuration [9,10]. The NIR luminescence performance of Cr^3+^ is highly dependent on the host structure, so the selection of the host material is of great significance for the realization of a stable and efficient NIR phosphor. The chemical formula of garnet structure is usually represented as A_3_B_2_C_3_O_12_, which belongs to the cubic crystal system with space group Ia-3d. Moreover, the garnet structure is an outstanding matrix candidate for Cr^3+^ due to its multiple cation sites and high structural rigidity [11,12]. Based on the above advantages, Cr^3+^-activated NIR luminescent materials with garnet matrix structures have received great attention in recent years, such as Ca_2_LuZr_2_Al_3_O_12_:Cr^3+^ [13], X_3_Sc_2_Ga_3_O_12_:Cr^3+^ (X = Lu, Y, Gd, La) [14], Lu_2_CaMg_2_Si_3_O_12_:Cr^3+^ [15], Y_3_Al_2_Ga_3_O_12_:Cr^3+^ [16], and Ca_3_Y_2_Ge_3_O_12_:Cr^3+^ [17], etc. In addition, thermal quenching has always been another challenge in manufacturing high-quality NIR pc-LEDs [18]. Therefore, it is necessary to develop more efficient and stable NIR phosphors.

In this study, we have successfully designed a highly efficient NIR luminescent phosphor, Ca_3_Mg_2_ZrGe_3_O_12_:Cr^3+^ (CMZG:Cr^3+^). The CMZG unit cell is a typical garnet structure. And the Ca atoms have [CaO_8_] polyhedral coordination, while Mg and Zr are coordinated by six oxygen atoms to form [MgO_6_] and [ZrO_6_] octahedron, and Ge atoms have [GeO_4_] tetrahedron coordination, respectively. The [CaO_8_] polyhedron, [MgO_6_] and [ZrO_6_] octahedron, and [GeO_4_] tetrahedron are interconnected in the CMZG matrix structure by sharing oxygen atoms. This study provides a detailed discussion of the ion-doping substitution structure, RT luminescence properties, and temperature-dependent luminescence behavior of this phosphor. By employing a Cr^3+^-Cr^3+^ dual-ion co-substitution strategy to replace Mg^2+^ and Zr^4+^ ions in CMZG, the resultant dual-site and aliovalent doping effectively optimizes NIR luminescence performance. Research indicates that Cr^3+^ ions, acting as chromophore ions, occupy both [MgO_6_] and [ZrO_6_] octahedra. The CMZG:Cr^3+^ phosphors exhibit a broad NIR emission spectrum with an FWHM of 89 nm and a peak wavelength of 758 nm. By fine-tuning the Cr^3+^ ion doping concentration, the optimal doping level was determined to be 1 mol%. The CMZG:0.01Cr^3+^ material retains excellent luminescent performance, even at temperatures as high as 250 °C, which shows negative thermal quenching characteristics. Additionally, the CMZG:0.01Cr^3+^ material shows promising application potential in night vision illumination. These findings provide a new avenue for the development and diversification of highly efficient, thermally stable NIR luminescent phosphors and lay a solid foundation for further applications.

## 2. Results and Discussion

### 2.1. Crystal Structure

**Figure 1:**
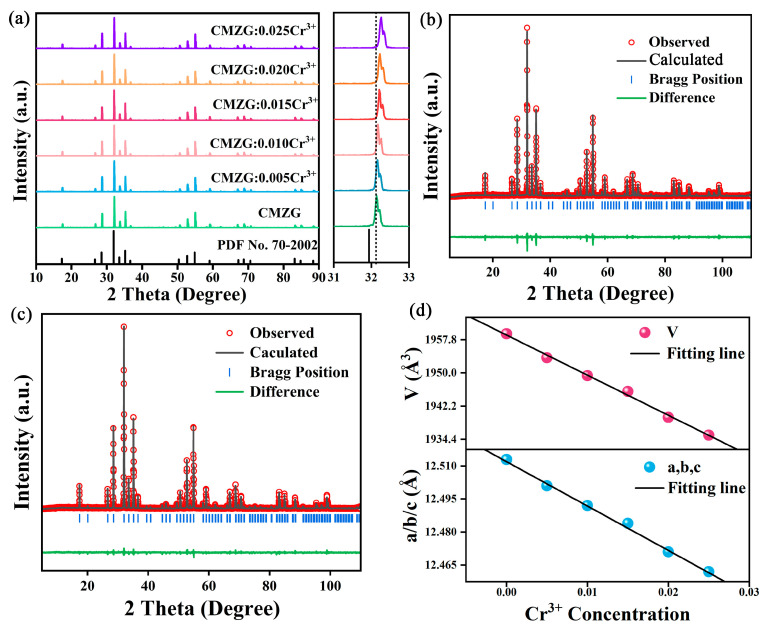
(**a**) XRD patterns of CMZG:xCr^3+^ (0.005 ≤ x ≤ 0.025); Rietveld refinement pattern of (**b**) CMZG; (**c**) CMZG:0.01Cr^3+^; and (**d**) lattice parameters and cell volumes of CMZG:xCr^3+^ (0.005 ≤ x ≤ 0.025).

Figure 1a depicts the powder X-ray diffraction (PXRD) patterns of the CMZG host, CMZG:xCr^3+^ (0.005 ≤ x ≤ 0.025), and the corresponding standard card. These whole diffraction peaks were well assigned to the PDF#70–2002 of CMZG, demonstrating that this series of samples are all pure phase and the introduction of Cr^3+^ ions does not affect the structural evolution. Therefore, the introduction of doped ions inevitably leads to ion substitution. By combining ion radius and valence state equilibrium, we preliminarily verified the feasibility of ion substitution through the acceptable percentage difference (*D_r_*) equation [19]:

(1) $$D_{r}=\frac{R_{m}(CN)-R_{d}(CN)}{R_{m}(CN)}$$

where *CN* represents the coordination number, *R_d_* represents the ionic radius of the dopant ion, and *R_m_* represents the ionic radius of the substituent ion. The following are the ionic radii of CMZG in different coordination environments: Mg^2+^ (0.72 Å, CN = 6), Zr^4+^ (0.72 Å, CN = 6), and Cr^3+^ (0.615 Å, CN = 6) [20]. From the calculation of Equation (1), the *D_r_* values of Mg^2+^ and Zr^4+^ replaced by Cr^3+^ were all 14.58%, indicating that Cr^3+^ can replace Mg^2+^ and Zr^4+^. The reliability of the feasibility of replacement was once again confirmed through XRD refinement. As shown in Figure 1b,c, the Rietveld refinements of the CMZG host and CMZG:0.01Cr^3+^ sample were carried out. The small fitting factor parameters (R_p_ = 9.7%, R_wp_ = 12.2%, χ^2^ = 1.83; R_p_ = 8.0%, and R_wp_ = 11.5%, χ^2^ = 1.94) imply that the refinement results are acceptable. More detailed refinement results are displayed in Table 1, which shows that the CMZG host and CMZG:xCr^3+^ (0.005 ≤ x ≤ 0.025) phosphors crystallized in the cubic structure with the Ia-3d space group. By analyzing the refinement data of different Cr^3+^ ion doping levels (Figure 1d), it can be concluded that the lattice constants and cell volumes of the CMZG:xCr^3+^ (0.005 ≤ x ≤ 0.025) samples are inversely proportional to the doping level of Cr^3+^ ions, which once again verifies the possibility of Cr^3+^ ions replacing Mg^2+^ and Zr^4+^ ions.

**Figure 2:**
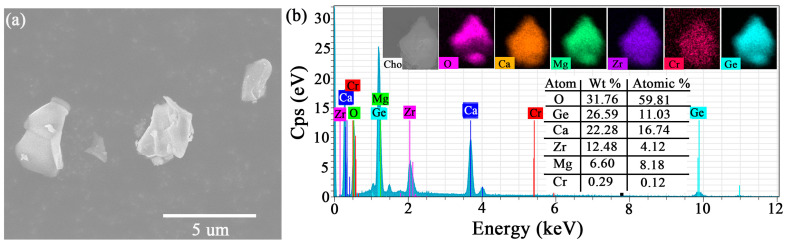
The (**a**) SEM and (**b**) elemental mapping image for the CMZG:0.01Cr^3+^ sample.

In order to further analyze the surface morphology, particle size, and distribution uniformity of doped elements (Cr^3+^) in the CMZG:0.01Cr^3+^ sample, we conducted scanning electron microscopy (SEM) morphology testing and energy dispersive spectroscopy (EDS) element distribution. As shown in Figure 2a, the particles of CMZG:0.01Cr^3+^ consist of many irregular single or clustered particles of about 2–5 µm in size. There is an obvious agglomeration phenomenon commonly seen in the solid-state method [21]. The micrometer size of the sample gives it great application potential in light-emitting devices. The EDS spectrum shown in Figure 2b depicts that the CMZG:0.01Cr^3+^ phosphor consists primarily of Ca, Mg, Zr, Ge, O, and Cr elements. The EDS element distribution test results of CMZG:Cr^3+^ phosphor indicate the successful doping of Cr^3+^ ions and their uniform distribution, as shown in the inset of Figure 2b. The table in the figure lists the weight percentage of each element, where the specific weight and atomic ratio of each element is close to the nominal one, indicating that the synthesis of phosphors was successful.

### 2.2. Optical Luminescence Properties

**Figure 3:**
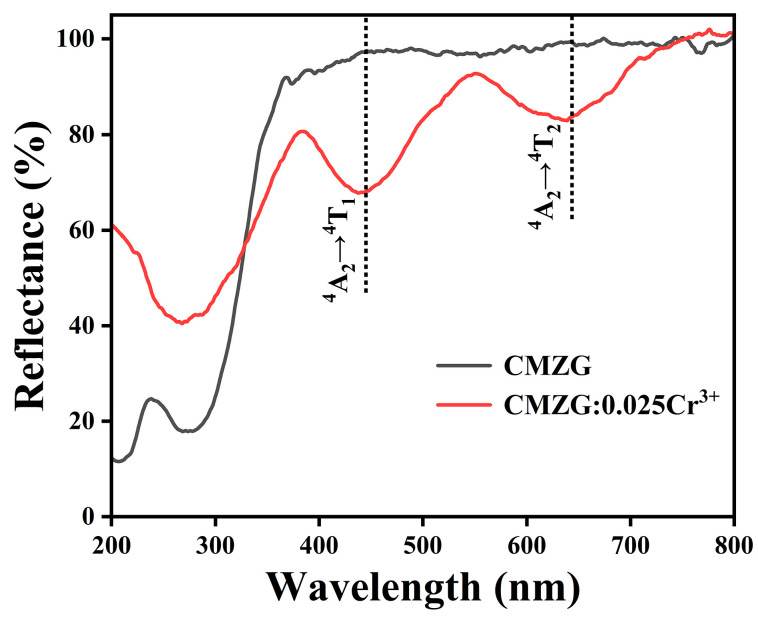
The diffuse reflectance spectra of the CMZG host and CMZG:0.025Cr^3+^ phosphor.

In order to understand the energy level structure of Cr ions and determine the absorption of Cr^3+^ ion-doped phosphors at different wavelength ranges, Figure 3 shows the diffuse reflectance spectra of the CMZG host and CMZG:0.025Cr^3+^ phosphor. As for the Cr^3+^-doped CMZG phosphors, the CMZG:0.01Cr^3+^ and CMZG:0.025Cr^3+^ samples exhibit significant absorption characteristics in the ultraviolet-to-visible region, especially strong broad absorption peaks in the range of 350 nm to 800 nm, which are similar to reported Cr^3+^-doped single crystals, such as Y_3_Al_5_O_12_:Cr^3+^ and Ca_3_NbGa_3_Si_2_O_14_:Cr^3+^ [22,23]. These broad absorption bands can be ascribed to ^4^A_2_→^4^T_1_ and ^4^A_2_→^4^T_2_ energy level transitions from Cr^3+^ ions.

**Figure 4:**
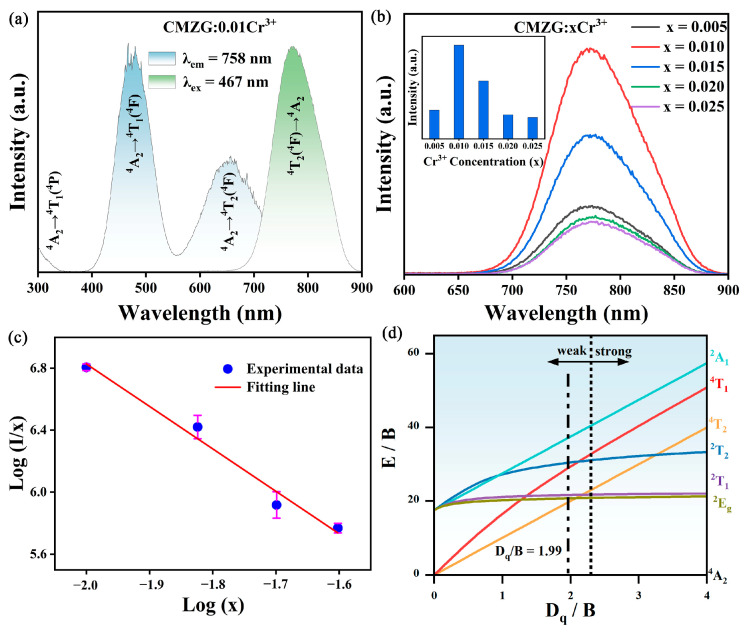
(**a**) The PLE and PL spectra of CMZG:0.01Cr^3+^ phosphor; (**b**) the PL spectra of CMZG:xCr^3+^ (0.005 ≤ x ≤ 0.025) phosphors; (**c**) linear fit of lg(*x*) vs. lg[*I*/(*x*)]; and (**d**) Tanabe–Sugano energy level diagrams for Cr^3+^.

Figure 4a shows the photoluminescence (PL)/PL excitation (PLE) spectra of the CMZG:0.01Cr^3+^ sample. Under 467 nm excitation, this phosphor exhibits a broadband NIR emission in the range of 600 to 900 nm with a primary emission peak at 758 nm, corresponding to the spin-allowed ^4^T_2_→^4^A_2_ transition of Cr^3+^ ions and a FWHM of 89 nm. By monitoring the emission at 758 nm, the PLE spectrum of this phosphor revealed two main excitation bands with peak wavelengths at 467 nm and 657 nm, corresponding to the typical ^4^A_2_→^4^T_1_ and ^4^A_2_→^4^T_2_ transitions of Cr^3+^ ions, respectively [24]. It is worth noting that the main excitation peak at 467 nm was in the blue light range, indicating that the CMZG:Cr^3+^ phosphor can achieve efficient NIR emission through excitation by blue LED chips. The strong excitation response to blue LED chips makes the CMZG:Cr^3+^ phosphor widely applicable in various practical applications, including non-destructive analysis, night vision, and biomedical imaging. As observed in Figure 4b, by increasing the Cr^3+^ concentration, the Cr^3+^ emission intensities (^4^T_2_ (F)→^4^A_2_ transition) first increased and then decreased as a result of concentration quenching [25]. When the doping concentration was 0.01, the intensity was at its maximum value. The intrinsic mechanism underlying the concentration quenching phenomenon of CMZG:xCr^3+^ (0.005 ≤ x ≤ 0.025) phosphors is further explored and characterized in Figure 4c. The critical distance (*R*_c_) in CMZG:xCr^3+^ (0.005 ≤ x ≤ 0.025) phosphors can be fitted using Blasse’s formula [26]:

(2) Rc=2(3V4πxcZ)13

where *V* represents the volume of the host lattice unit cell, *Z* represents the number of available sites for Cr^3+^ ions, and xc is the optimum luminous concentration. For CMZG, *Z* is 8, *x_c_* is 0.01, *V* is 1959.22 Å^3^, and the *R*_c_ is calculated to be 36 Å, which corresponds to the multipole–multipole interaction. Based on the Dexter theory, we conducted further mechanism research and analyzed the linear fitting relationship between log(*I/x*) and log(*x*) using the following formula [27]:

(3) $$\frac{I}{x}=K{\left(1+\beta {\left(x\right)}^{Q/3}\right)}^{-1}$$

where *x* denotes the concentration, *I* denotes luminescence intensity, and *K* and *β* are substrate-dependent constants. The *Q* values of 6, 8, and 10 correspond to dipole–dipole interactions, dipole–quadrupole interactions and quadrupole–quadrupole interactions, respectively [28]. As shown in Figure 4c, the slope can be obtained by linear fitting as −2.74, so the value of Q for CMZG:xCr^3+^ (0.005 ≤ x ≤ 0.025) is calculated as 8.19, which suggests that the concentration quenching mechanism of CMZG:xCr^3+^ is caused by dipole–quadrupole interactions.

It is well-known that the 3D orbital electrons of Cr^3+^ ions are not shielded by the outer shell and its surroundings, so the photoluminescence of Cr^3+^ is extremely sensitive to the crystal field environment of the main lattice [29]. When Cr^3+^ occupies the octahedral position (O_h_ point group), its energy level variation with the crystal field strength can be described by the Tanabe–Sugano diagram, as depicted in Figure 4d, which is calculated as follows [30]:

(4) Dq=E(A2g4→T2g4)/10

(5) $$\frac{D_{q}}{B}=\frac{15(x-8)}{x^{2}-10x}$$

(6) x=E(A2g4→T1g4)−E(A2g4→T2g4)Dq

where *D_q_* is the crystal field parameter, *B* represents the Racah parameter, and the value of *D_q_/B* has a great influence on the crystal field and affects the emission bandwidth of Cr^3+^. When *D_q_*/*B* < 2.3, the ^4^T_2_→^4^A_2_ transition of Cr^3+^ dominates, and Cr^3+^ occupies a weak crystal field and shows broadband emission. In contrast, when *D_q_*/*B* > 2.3, Cr^3+^ occupies a strong crystal field and exhibits a narrowband emission corresponding to the ^2^E→^4^A_2_ transition [31]. Based on the above theoretical calculations, it is known that the values of *D_q_* and *B* for the CMZG:0.01Cr^3+^ sample are 1319 cm^−^^1^ and 663 cm^−^^1^, respectively, and, thus, the *D_q_*/*B* value is 1.99, which is less than 2.3, suggesting that Cr^3+^ occupies Mg^2+^ and Zr^4+^ lattice sites with a weak crystalline field in the CMZG matrix, which is consistent with the broadband emission characteristic of this sample at room temperature.

**Figure 5:**
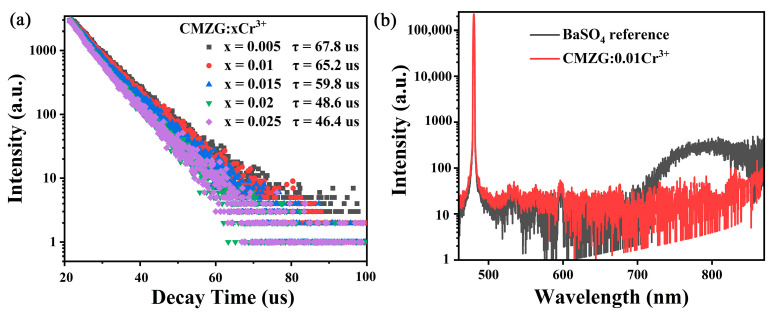
(**a**) Luminescence decay curves of CMZG:xCr^3+^ (0.005 ≤ x ≤ 0.025) phosphors; (**b**) excitation line of BaSO_4_ and emission spectrum of CMZG:0.01Cr^3+^ phosphor.

The evaluation of the phosphor’s lifetime characteristics is crucial for understanding its optical properties. By measuring the lifetime characteristics of the phosphor, we can guide its application development in optoelectronic devices and other fields. Figure 5a shows the luminescence decay curves of CMZG:xCr^3+^ (0.005 ≤ x ≤ 0.025) phosphors at RT monitored under excitation at 467 nm and emission at 758 nm. The average lifetimes (*τ*) are calculated using the following equation [32,33]:

(7) $$\tau =\frac{\int_{0}^{\infty }tI(t)dt}{\int_{0}^{\infty }I(t)dt}$$

where *I*(*t*) represents the PL intensity at time t. It can be inferred that the average decay time values of CMZG:xCr^3+^ (0.005 ≤ x ≤ 0.025) samples are 67.8 µs, 65.2 µs, 59.8 µs, 48.6 µs, and 46.4 µs. Additionally, with the increase in Cr^3+^ doping concentration, the fluorescence lifetime of the phosphor gradually decreases from 67.8 μs to 46.4 μs. These results indicate that a noticeable energy transfer occurred between Cr^3+^ ions in the CMZG:xCr^3+^ (0.005 ≤ x ≤ 0.025) phosphors, which could easily lead to concentration quenching [34]. The evaluation of internal quantum efficiency (IQE) has significance for the practical application of CMZG:Cr^3+^ phosphors. Therefore, the IQE spectrum of the CMZG:0.01Cr^3+^ sample measured at RT under excitation at 467 nm is shown in Figure 5b, and the IQE for the CMZG:0.01Cr^3+^ phosphor can be obtained by the following equation [35]:

(8) $$\eta =\frac{\int L_{s}}{\int E_{R}-\int E_{s}}$$

where ∫L_S_ is the integral area of the emission spectrum, and ∫E_R_ and ∫E_S_ are the integral areas of the excitation spectra without and with phosphor, respectively. And the IQE value of the CMZG:0.01Cr^3+^ sample reaches up to 68.7%, which is higher than recently reported Cr^3+^-doped phosphors such as Mg_3_Ga_2_GeO_8_:Cr^3+^ (35%) [7], La_2_LiSbO_6_:Cr^3+^ (62.4%) [10], Lu_3_Sc_2_Ga_3_O_12_:Cr^3+^ (60%) [36], La_3_Ga_5_SnO_14_:Cr^3+^ (42.7%) [37], and La_3_Ga_5_SiO_14_:Cr^3+^ (62.3%) [38]. According to previous reports, the high IQE of the CMZG:0.01Cr^3+^ phosphor occupies a high level in the oxide fluorescent powder system, which is beneficial for its practical application.

### 2.3. Thermal Stability

**Figure 6:**
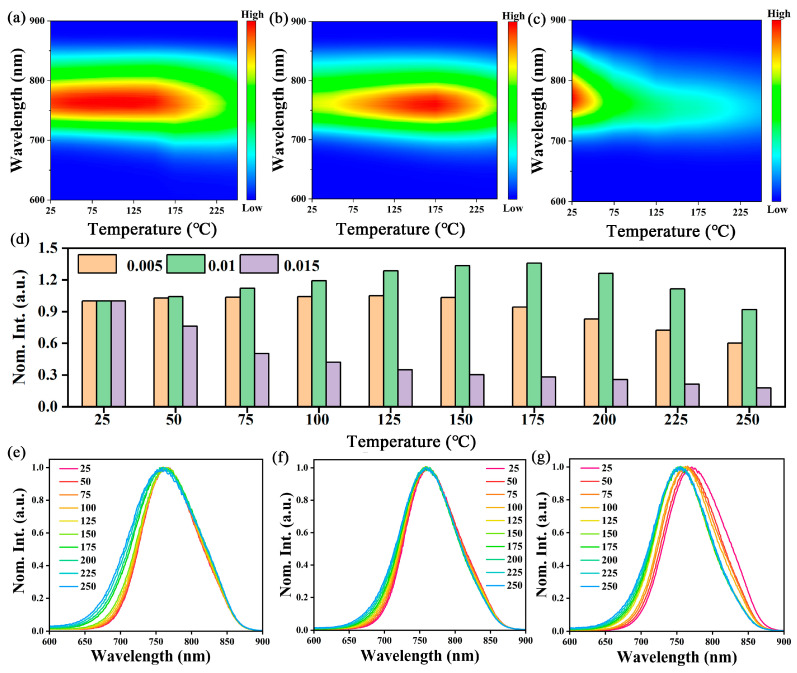
Temperature-dependent PL spectra of (**a**) CMZG:0.005Cr^3+^ (**b**) CMZG:0.01Cr^3+^ and (**c**) CMZG:0.015Cr^3+^ phosphors; (**d**) temperature-dependent integral emission intensity of these phosphors; and normalized temperature-dependent PL spectra of (**e**) CMZG:0.005Cr^3+^ (**f**) CMZG:0.01Cr^3+^, and (**g**) CMZG:0.015Cr^3+^ phosphors.

The fluorescence thermal quenching performance of phosphors for LEDs is an important index to judge whether this method can be applied, especially for NIR phosphors, whose fluorescence thermal effect is more significant than that of visible phosphors due to the large Stokes shift [39]. Therefore, it is important to understand the thermal quenching properties of phosphors to develop highly stable NIR phosphors. The temperature-dependent PL spectra of CMZG:0.005Cr^3+^, CMZG:0.01Cr^3+^, and CMZG:0.015Cr^3+^ phosphors tested in the temperature range of 25~250 °C are shown in Figure 6a–c. Clearly, the waveforms and emission peak positions of the emission spectra of the samples at different temperatures are basically the same. The relationship between the normalized luminescence intensity of the three samples and the temperature is given in Figure 6d, and the luminescence intensity of the three materials shows a different pattern as the temperature increases. CMZG:0.015Cr^3+^ shows a very rapid decrease in intensity as the temperature increases due to the temperature quenching effect, while CMZG:0.005Cr^3+^ and CMZG:0.01Cr^3+^ show an increase and then a decrease in intensity as the temperature increases. The CMZG:0.01Cr^3+^ sample shows the best thermal stability: the luminous intensity can reach up to about 133% at 150 °C compared with room temperature, and even at 250 °C, the intensity remains at 92% of the room temperature intensity. The anti-thermal quenching phenomenon was observed in the CMZG:0.01Cr^3+^ phosphor. According to previous reports, lattice defects can act as electron-trapping centers that store and transfer energy to Cr^3+^ excited levels, releasing trapped electrons as NIR photons with increasing temperature and thereby enhancing NIR PL intensity [40,41]. This result is higher than almost all known Cr^3+^-activated NIR phosphors, as shown in Table 1. The normalized intensity plots of the CMZG:0.005Cr^3+^, CMZG:0.01Cr^3+^, and CMZG:0.015Cr^3+^ samples at different temperatures are displayed in Figure 6e–g. It is obvious that the best peak position of CMZG:0.001Cr^3+^ changes less with increasing temperature compared to the other two materials, which further indicates that it has better luminescence stability.

**Table 1:** Recent thermal stability of Cr^3+^-activated phosphors.

Sample	Thermal Stability at 423 K	*E_a_* (eV)	Ref
Gd_3_Zn_0.8_Ga_3.4_Ge_0.8_O_12_:Cr^3+^	40.2%	0.324	[11]
Ca_2_LuScGa_2_Ge_2_O_12_:Cr^3+^	59%	0.17	[18]
Lu_2_CaAl_4_SiO_12_:Cr^3+^	66.2%	0.28	[27]
Lu_2_CaMg_2_Si_3_O_12_: Cr^3+^	70%	0.38	[15]
Y_3_Sc_2_Al_3_O_12_:Cr^3+^	97%	0.21	[42]
CMZG:0.01Cr^3+^	133%	0.48	This work

**Figure 7:**
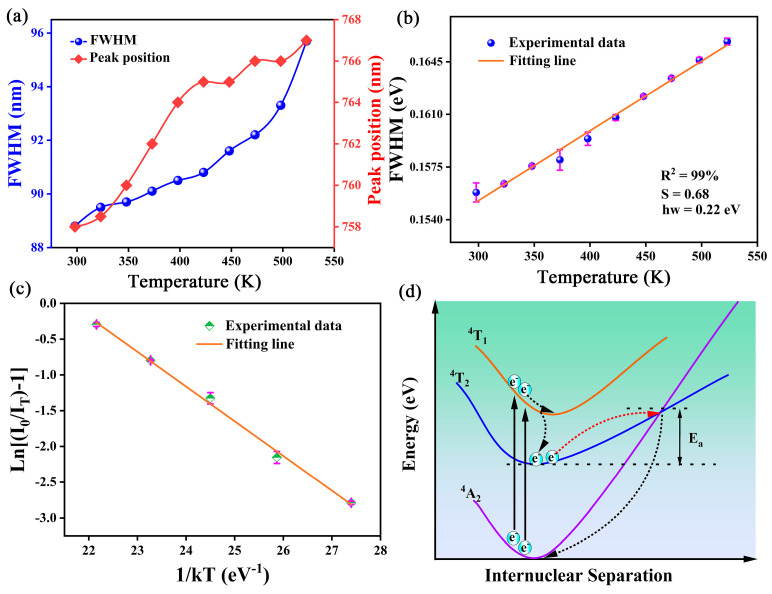
(**a**) Variations in the emission peak and FWHM with increasing temperature; (**b**) fitted Huang–Rhys factor and phonon energy; (**c**) the dependence of ln[(*I*_0_/*I_T_*) − 1] on 1/*kT* for the CMZG:0.01Cr^3+^ sample; and (**d**) configurational coordinate diagram of Cr^3+^; (The red dashed line represents the transition process generated by heating and the black dashed line represents the non-radiative transition).

As shown in Figure 7a, the CMZG:0.01Cr^3+^ sample exhibited different FWHM at various temperatures, corresponding to Cr^3+^ ions occupying Mg and Zr sites. Compared to other Cr^3+^-activated luminescent materials and reported studies in the literature on Cr^3+^ ion luminescence, a broader FWHM indicates weaker electron–phonon coupling for Cr^3+^ ions in the CMZG host. Moreover, the spectra are broadened from 88.8 nm to 95.7 nm with increasing temperature as a result of the reduced crystal field strength and the increased electron–phonon coupling effect of Cr^3+^ electrons [43]. As shown in Figure 7b, based on the functional relationship between FWHM and temperature, we performed a linear fit using Equation (9) [44]:

(9) $$FWHM=2.36\sqrt{S}\hbar \omega \sqrt{\coth\frac{\hbar \omega }{2kT}}$$

where ℏ*ω* is the effective phonon energy that interacts with the electronic transitions, *T* is the Kelvin temperature, *S* is the dimensionless Huang–Rhys factor, and *k* is the Boltzmann constant (0.8617 × 10^−5^ eV). The fitting results indicate that the value of the *S* is 0.68, and a very small value indicates that the electron–photon coupling effect in CMZG:Cr^3+^ is relatively weak and can be almost ignored [45]. The very weak electron–photon coupling effect promotes the CMZG:Cr^3+^ sample to exhibit a relatively small Stokes shift compared to other Cr^3+^-doped phosphors. However, at the same time, a relatively small Stokes shift also means a larger thermal activation energy, resulting in relatively weak fluorescence thermal quenching. This conclusion is further demonstrated in Figure 7c. The activation energy (*E_a_*) can be determined by the fitting relationship between ln[(*I*_0_/*I_t_*) − 1)] and 1/*KT* using the following equation [46]:

(10) $$I(T)=\frac{I_{0}}{1+c\exp(-(E_{a}/KT))}$$

Using Formula (10), a linear relationship between ln[(*I*_0_/*I_t_*) *−* 1)] and 1/*KT* can be obtained. The final fitting result is shown in Figure 7c, and the fitting value of *E_a_* that is obtained by calculating the slope is 0.48 eV. As shown in Table 1, this result is higher than the recently reported NIR phosphors. Usually, we can explain the thermal quenching phenomenon with the bitmap coordinate model depicted in Figure 7d. When the electrons of Cr^3+^ in the ^4^A_2_ energy level are excited by blue light, they jump to the ^4^T_2_ energy level, and part of the electrons jump from the bottom of the ^4^T_2_ energy level back to the ^4^A_2_ energy level and emit NIR light; the other part of the electrons jump to the intersection of the ^4^T_2_ and the ^4^A_2_ energy levels under thermal excitation, and then return to the ^4^A_2_ energy level through a non-radiative transition [47]. Therefore, the wide activation energy, the weak electron–photon coupling effect, and the presence of defect traps contribute greatly to the remarkable anti-thermal quenching properties of CMZG:0.01Cr^3+^.

### 2.4. Potential Applications

**Figure 8:**
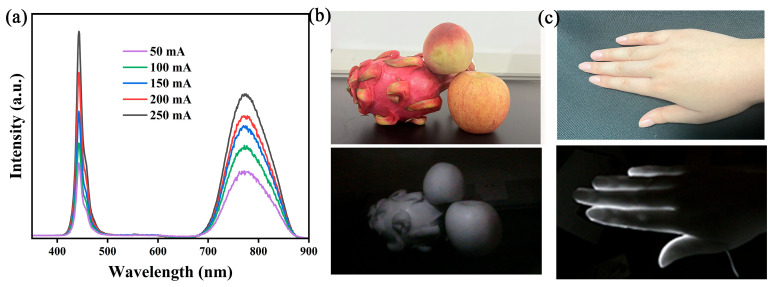
(**a**) The EL spectra of fabricated NIR pc-LEDs under various driving currents; (**b**) photographs of NIR light penetrating a palm; and (**c**) images taken under fluorescent light and NIR LED light.

To evaluate the performance of CMZG:0.01Cr^3+^ NIR phosphors in NIR devices and demonstrate their valuable potential applications, we mixed CMZG:0.01Cr^3+^ phosphors with a specialized encapsulation adhesive and coated them onto a 450 nm commercial blue InGaN chip, packaging it into an NIR pc-LED device, as shown in the inset of Figure 8a. The electroluminescence (EL) spectra of the prepared device under different driving currents are shown in Figure 8a. As the driving current increases, the luminous intensity increases, indicating that the device has good current stability [48]. Furthermore, utilizing the strong tissue penetration and non-destructive characteristics of NIR light from this device, we applied the packaged NIR pc-LED device to palm vein imaging, as illustrated in Figure 8b. By covering the palm over the illuminated pc-LED device and taking a photograph with a NIR camera, the blood vessels in the palm could be clearly seen as a result of the stronger absorption of NIR light by the blood vessels. Figure 8c shows the prepared pc-LED device for night vision illumination application. The upper figure shows an image of fruit under fluorescent light irradiation using a visible camera, and the lower figure shows an image taken by a near-infrared camera under pc-LED irradiation. As can be seen from the figure, the NIR image is clearly visible, demonstrating its value for night vision lighting applications. These results indicate that CMZG:Cr^3+^ phosphors have potential application value in biological tissue imaging and non-destructive testing.

## 3. Materials and Methods

### 3.1. Preparation of Materials

The samples of the CMZG:xCr^3+^ phosphors were prepared by the high-temperature solid-state method using proper amounts of Calcium Carbonate (CaCO_3_, 99.5%), Magnesium Oxide (MgO, 99.5%), Zirconium Oxide (ZrO_2_, 99.5%), Germanium Oxide (GeO_2_, 99.99%), and Chromium (III) Oxide (Cr_2_O_3_, 99.99%) as initial materials and no further purification was required. For the CMZG host, 3 mmol CaCO_3_ (0.3000 g), 1 mmol MgO (0.0403 g), 1 mmol ZrO_2_ (0.1232 g), and 3 mmol GeO_2_ (0.3138 g) were added to the agate mortar for thorough grinding. For the Cr^3+^-doped CMZG phosphors, MgO and ZrO_2_ were replaced in the matrix with an equal amount of Cr_2_O_3_, and the grinding process was the same. Subsequently, the above mixture was transferred to multiple crucibles and placed in the middle of a muffle furnace for high-temperature (1400 °C) calcination under an air atmosphere for 6 h. After the high-temperature process was completed, the mixture was removed and ground to obtain CMZG:xCr^3+^ phosphors.

### 3.2. Characterization of Materials

Using PXRD (Bruker D8 Advance, Billerica, MA, USA) with a monochromatized source of Cu-Kα radiation (λ = 0.15406 Å) at 40 kV and 30 mA (2θ = 10–90°, PXRD data; 2θ = 5–110°, Rietveld refinement data), the PXRD and Rietveld refinement data of the CMZG:xCr^3+^ phosphors were characterized. The Rietveld structures of the CMZG host and CMZG:xCr^3+^ phosphors were fitted and analyzed by Fullprof software (Windows 64 bits, July-2011). The SEM images and EDS analysis were detected using Hitachi S-4800 equipment (SEM, Tokyo, Japan). FLS1000 spectrophotometers (Edinburgh, Livingston, UK) were utilized to survey the PL and PLE diagrams and the decay curves at RT. The measurement results of temperature-dependent luminescence were also collected using the FLS1000 fluorescence spectrophotometer (Edinburgh, Livingston, UK), which was equipped with a fixed-power Xenon lamp. The RT IQE data of CMZG:0.01Cr^3+^ were analyzed using this spectrophotometer equipped with the matching integrating sphere device.

## 4. Conclusions

In summary, we successfully prepared a series of Cr^3+^-doped CMZG phosphors using the high-temperature solid-state method with micron-level particle size. Under the blue light excitation at 467 nm, these phosphors exhibited an emission at 758 nm with an FWHM of 89 nm. Due to the doping strategy and the unique multi-substitution lattice crystal structure of the main lattice of CMZG, a high IQE of 68.7% was achieved. Using spectral data, the crystal field strength *D_q_*/*B* of Cr^3+^ in the CMZG matrix was calculated to be 1.99. Due to the relatively weak electron–phonon coupling, the CMZG:0.01Cr^3+^ NIR phosphor exhibited a relatively small Stokes shift in the PL spectra. This also resulted in notable thermal stability, with the luminescence intensity at 250 °C being approximately 92% of that at RT. Our results provide an auspicious NIR material for investigating NIR-emitting phosphors for biological tissue imaging and non-destructive applications.

## Data Availability

More research data are available from the authors upon request.
